# Optimization of T Cell Redirecting Strategies: Obtaining Inspirations From Natural Process of T Cell Activation

**DOI:** 10.3389/fimmu.2021.664329

**Published:** 2021-04-26

**Authors:** Yiyuan Gao, Yuedi Wang, Feifei Luo, Yiwei Chu

**Affiliations:** ^1^ Institutes of Biomedical Sciences, and Department of Immunology, School of Basic Medical Sciences, Fudan University, Shanghai, China; ^2^ Biotherapy Research Center, Fudan University, Shanghai, China; ^3^ Department of Digestive Diseases, Huashan Hospital, Fudan University, Shanghai, China

**Keywords:** chimeric antigen receptor, bispecific antibody, immunological synapse, metabolism, T cell-based immunotherapy

## Abstract

Chimeric antigen receptors (CARs) or bispecific antibodies (bsAbs) redirected T cell against tumors is one of the most promising immunotherapy approaches. However, insufficient clinical outcomes are still observed in treatments of both solid and non-solid tumors. Limited efficacy and poor persistence are two major challenges in redirected T cell therapies. The immunological synapse (IS) is a vital component during the T cell response, which largely determines the clinical outcomes of T cell-based therapies. Here, we review the structural and signaling characteristics of IS formed by natural T cells and redirected T cells. Furthermore, inspired by the elaborate natural T cell receptor-mediated IS, we provide potential strategies for higher efficacy and longer persistence of redirected T cells.

## Introduction

Redirecting T cell toward tumors under the assistance of chimeric antigen receptors (CARs) or bispecific antibodies (bsAbs) has exhibited unprecedented antitumor capacity in cancer treatments (1–3). However, the redirected T cells are faced with challenges of limited efficacy and poor persistence, which lead to a high rate of relapse after treatments for both solid and non-solid tumors, and severely inhibit the broader application of redirected T cell-based therapy (3–6).

The immunological synapse (IS) acts as a core mechanism of the T cell response, by delivering activations signals and releasing lytic granules (7–10). The metabolic state of T cell will quickly adjust to favor the subsequent immune response after its activation (11). Not only T cell receptor (TCR), but also several structural and signaling molecules are involved in IS formation and stability, which has been thoroughly explored (7, 12–16), but few investigations focus on IS generated by CAR and bsAb. The absence of some structural and signaling molecules in CAR/bsAb design might result in the unstable characteristics of CAR-/bsAb-mediated IS, and further hinder redirected T cell activation and function. Therefore, generation of a high-quality IS with stable structure and sustainable signaling could be an ideal way to enhance the efficacy and prolong the persistence of redirected T cells. Here, we review the structural and signaling characteristics of IS formed by natural T cells and redirected T cells. Inspired by the advantages exhibited in natural TCR-mediated activation, we provide potential strategies for higher efficacy and longer persistence of redirected T cells by emulating the structure and signaling of natural TCR-IS.

## Structural Features in Natural IS

The IS is a stable interface between antigen-presenting cell (APC) and T cell organized by orchestrated rearrangement of diverse signaling and structural molecules (8). There are multiple types of IS. For example, CD4^+^ T cells connect with APCs and generate the classical IS, which can stimulate T cell activation (17); CD8^+^ T cells form the cytolytic IS, which triggers lytic granules releasing and the destruction of tumor cells (17, 18).

**Figure 1:**
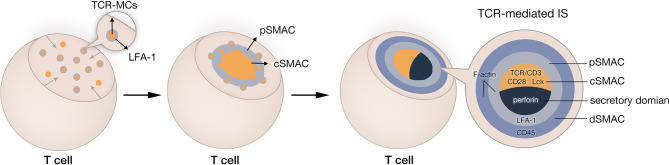
Natural IS formation in cytotoxicity T lymphocyte. After recognition of tumor cell, TCR-CD3 complexes aggregate and form TCR microclusters (TCR-MCs). TCR-MCs contain various TCR downstream signaling molecules and adhesion molecules. These TCR-MCs move toward the center of the interface between T cell and its target and form the central supramolecular activation cluster (cSMAC). In this process, adhesion molecule LFA-1 dissociates from TCR-MCs and remains in the peripheral region of the IS, forming the peripheral SMAC (pSMAC). The outmost ring of immune synapse is distal SMAC (dSMAC).

TCR-IS known as a bull’s eye structure, is a three-layered concentric dynamic structure (**Figure 1**). It is composed of the central supramolecular activation cluster (cSMAC), the peripheral SMAC (pSMAC), and the distal SMAC (dSMAC) (8). The formation of IS is a complicated and elaborate process. Taking cytolytic IS as an example, T-cell activation signaling followed by antigen recognition is amplified by transiently engaged TCR-CD3 complex, called TCR microclusters (TCR-MCs) (19). These TCR-MCs contain a diverse range of signaling proteins, including costimulatory receptors (such as CD28), downstream signaling proteins, and adhesion molecules (8, 20, 21). Under the assistance of cytoskeleton proteins, TCR-MCs then translocate toward the center of the cell-cell interface, forming the cSMAC (8, 22). During TCR-MCs centripetal movement, the adhesion integrin leukocyte function-associated antigen 1 (LFA-1) dissociates from TCR-MCs and surrounds the cSMAC in the form of a peripheral ring, defined as pSMAC (23, 24). The outmost ring of TCR-IS, known as dSMAC, contains various large and bulky molecules, such as CD43 and CD45 (25, 26). The abundant filamentous actin (F-actin) in pSMAC and dSMAC is essential for a stable IS and sustained activation signaling (27, 28). Additionally, the microtubule-organizing center (MTOC) is reoriented toward the IS, which allows the relocation of T-cell secretory domain and a polarized degranulation of lytic granules (29). Given that the IS simultaneously undertakes the responsibility for T-cell activation and cytolytic functionality, the outcome of T cell response is greatly influenced by the quality of the IS.

## Structural Features in CAR-/bsAb-Mediated IS

**Figure 2:**
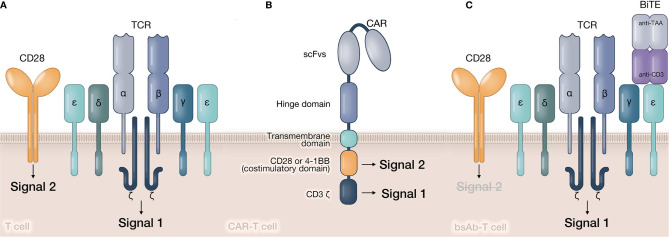
Structures of signaling transduction triggered by TCR, CAR, and bsAb. **(A)** In natural T cell, tumor antigen peptide is presented by APC and recognized by TCR. The associated CD3 molecule in TCR-CD3 complex will provide Signal 1 to T cell. The costimulatory signal (Signal 2) is provided by costimulatory molecules, such as CD28, 4-1 BB, etc. **(B)** In CAR-T cell, target antigens are directly recognized by CAR molecule’s scFv. Signal 1 and Signal 2 are provided by CD3ζ and costimulatory domain relatively in CAR’s intracellular domain. **(C)** In bsAb-T cell, bsAbs simultaneously recognize tumor antigen and CD3ϵ chain, and form a bridge between tumor cell and T cell. Similar to natural T cell, Signal 1 is still provided by TCR-CD3 complex. However, Signal 2 is lack in bsAb-mediated activation.

CARs and bsAbs assist T cells in bypassing the MHC restriction and simultaneously build bridges (forming immunological synapses) between T cells and tumor cells. CARs are synthetic transmembrane receptors that combine an antigen recognition single-chain fragment variable (scFv) with one or two signal transduction intracellular domains (**Figure 2**). Their designs are exactly based on the principle of conventional TCR signaling. Sequences from CD3ζ signaling domain and costimulatory molecules (such as CD28 or 4-1BB) respectively provide Signal 1 and Signal 2 for T cell activation (30). T-cell redirecting bsAbs are soluble artificial molecules that can simultaneously target the CD3ϵ chain of the TCR/CD3 complex and the tumor-associated antigen (31) (**Figure 2**). Thus, bsAb-mediated T cell activation is merely triggered by Signal 1. Several types of bsAbs with various sizes and formats have been created, including bsAbs with active Fc domain (32) and bsAbs without Fc domain but have different connecting modes, such as diabodies (33, 34), bispecific T cell engagers (BiTEs) (35, 36). The structural diversity of bsAbs can directly influence their linking efficacy and *in vivo* half-life, which will further determine the therapeutic effect of bsAbs (37, 38). Therefore, CAR- and bsAb-mediated ISs have obvious distinctions with natural TCR-IS in structural and signaling features. These characteristics are closely related to the limited clinical efficacy of redirected T cells.

### CAR-Mediated IS

**Figure 3:**
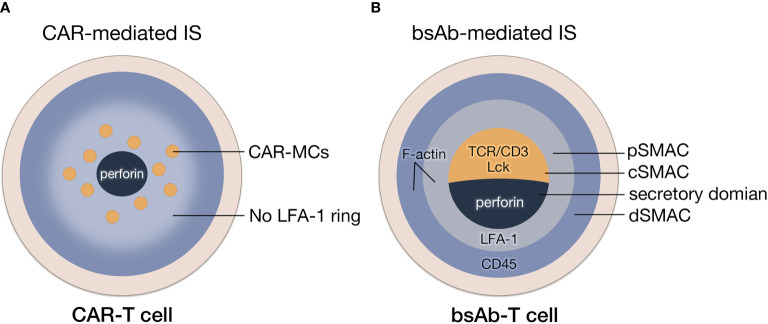
The formation of IS mediated by CAR and bsAb. **(A)** The structure of CAR-IS. The microclusters of CAR are dispersed in a multipolar manner surrounded by disorganized LFA-1 without a clear boundary of pSMAC. **(B)** The structure of bsAb-IS. Similar to TCR-IS, BsAb-IS has a conventional mature IS structure, with organized cSMAC, and a LAF-1 ring and actin accumulation at the periphery.

There is a clear structural distinction between CAR-mediated IS (CAR-IS) and TCR-IS. Although similar to TCR clustering, the initiation of activation signaling is triggered by engaged CARs (**Figure 3A**), much fewer molecules are involved in CAR clusters, which could lead to a spatiotemporal disorder in CAR-IS formation. For example, due to the lack of LFA-1 participation, there is no obvious boundary of pSMAC in CAR-IS (15, 39). CAR clusters are dispersed *via* a multipolar manner in the center of IS. Since the LFA-1 directed F-actin remodeling can strengthen the IS (40), it can be speculated that the stability of CAR-IS without LFA-1 is very limited. For instance, CAR-IS has a smaller size and faster formation than TCR-IS. Also, CAR-T cells have a faster detachment rate from the target cell than natural T cells (15). The phenomenon above should be a result of the absence of adhesion molecules in CAR-IS, which might lead to a weak cell-cell connection and aberrant signal transduction to induce T cell exhaustion. Additionally, CAR-IS mediated lytic granule secretion happens before MTOC polarization (41), indicating CAR-T-cell inadequate cytoskeleton remodeling. In conclusion, the rapid target cytolysis and poor persistence features of CAR-T cells may result from the instability of CAR-IS.

### BsAb-Mediated IS

As bsAb-mediated T-cell activation through crosslinking TCR-CD3 complexes, bsAb-mediated IS (bsAb-IS) is quite similar to TCR-IS. BsAb-IS has a conventional mature IS structure, with an organized cSMAC and a clear boundary of pSMAC (42) (**Figure 3B**). It has been reported that an anti-FcRH5/CD3 bispecific antibody stimulated T cell activation by inducing clustering and excluding CD45 phosphatase from the synapse displays a similar mechanism of the TCR/pMHC interaction mediated by TCR (16). In a phase 3 trail, patients with heavily pretreated B-cell precursor acute lymphoblastic leukemia received blinatumomab (an anti-CD19 BiTE) or chemotherapy (43). The median overall survival of blinatumomab group (7.7 months) was significantly longer than chemotherapy group (4.0 months), which exhibited the superior antitumor activity of bsAbs (43). However, the remission rates of blinatumomab within 12 weeks was only 44% (43). After 6-month treatment with blinatumomab, only 31% patients were event-free, which might be result from the poor persistence of blinatumomab-induced T cell response. (43). Considering bsAbs trigger CD3-induced T cell activation, the absence of Signal 2 during bsAb-mediated T-cell activation might lead to the poor persistence of bsAb-T cell function.

## Strategies for Optimization of Redirected T-cell Therapies

### Imitation of Natural IS Morphological Features

#### Involvement of Structural Molecules

Structural molecules, such as adhesion molecule LFA-1 and cytoskeleton proteins are essential for IS stability. The engagement of LFA-1 facilitates TCR-pMHC interaction and consolidates the formation of pSMAC. In an LFA-1 engagement absent situation, T cell sensitivity to antigen had a 100-fold decrease (44). LFA-1 also intimately interacts with cytoskeleton proteins in natural TCR-IS (14, 45). F-actin flow drives LFA-1 conformation change and leads to the formation of the LFA-1 ring (45). Meanwhile, LFA-1 favors T cell activation *via* participating in various T-cell signaling pathways, such as Notch pathway (46) and Erk1/2 signal pathways (47). The blockade of LFA-1 restrained the killing function of cytotoxic T cells (48, 49). Therefore, the absence of LFA-1 ring in CAR-IS could impact IS stability through the prevention of cytoskeleton protein participation and further hinder CAR-T cell MTOC polarization (47).

The actin cytoskeletal network helps orchestrate CAR clustering and plays a vital role in signal transduction. It has been reported that the utility of actin-polymerization inhibitors prevented CAR clustering (50). Mutated signaling-deficient CARs with inactivated intracellular domain still could form clusters in an actin-dependent manner (50). The quantitation of F-actin is an important factor to evaluate the quality of CAR-IS, which can be used to predict the effectiveness of CAR-modified immune cells (51, 52). It has been reported that 4-1BBz CAR-T cells exhibit a better F-actin accumulation than CD28z CAR-T cells, which could explain the superior tumor control from 4-1BBz CAR immunotherapy (52). Therefore, further improvement of CAR-IS’ structure, such as enhancing the stability of CAR-IS by involving adhesion molecules or cytoskeleton proteins, maybe a powerful strategy to strengthen CAR-T cell cytolytic function.

#### Increase of CARs/bsAbs Clustering

Natural TCR-MCs are crucial in amplifying initial T cell activation signals. CAR clusters and bsAb-mediated TCR-MCs also influence CAR-/bsAb-T cell activation and antitumor activity. A study found that green fluorescent protein (GFP)-binding CAR-T cells only responded to soluble GFP ligands with the ability of dimerizing CARs, which proved the importance of CARs dimerization in activation signaling (50). A tandem CAR-T cell targeting HER2 and IL13Rα2 forms an enhanced bivalent immune synapse by heterodimerizing its targets, which has a higher F-actin accumulation at the IS and increased MTOC polarization, making TanCAR-mediated IS with superior cytolytic potential (53). CAR’s bivalent interactions are especially meaningful to low-affinity CARs, which only triggered specific lysis with dimerization-promoting CD8α hinge (54). Hence, the hinge domain is an inescapable part of CAR design to increase CAR clustering. The hinge domain, usually derived from IgG (55–57), CD28 (58, 59), and CD8α (58–60), connects the scFv and the transmembrane (TM) domain in a CAR molecule. One case compared conventionally-used hinge and TM domains in CAR-T cells, suggesting that CAR’s clustering and signaling intensity were affected by the hinge domain (59). And CAR-T cells with CD3ζ, CD28 or CD8α hinge but not CD4 hinge would have enhanced signal transduction and superior *in vitro* response (59). Mechanically, CD3ζ, CD28 and CD8α hinges are cysteine-rich and originally generate dimers on T cells, while CD4 hinge has a monomer form in physiological state (59, 61–63). Thus, a potential improved approach is to design CAR with a cysteine-rich hinge such as 4-1BB and OX40 derived domains, which belong to the tumor necrosis factor receptor superfamily, and are naturally trimerized and abundant in cysteine (59, 64, 65).

It has been found that antagonistic anti-CD40 antibodies are able to be converted into potent FcγR-independent agonists by isotope switching to cystine-rich hIgG2 (66). The unique disulfide bonding properties in hIgG2 hinge enable CD40 clustering and activate the NF-kB signaling pathway, suggesting that hinge domain rich in cystine could also be used to optimize the signaling function of antibodies (66). Consistent with CAR molecules clustering to enlarge CAR-T cell functionality, increase of bsAbs-mediated TCR-MCs by using cystine-rich linker may offer a new direction for optimizing clinically-used bsAbs.

### Emulation of Natural IS Signaling Features

#### Complement of Full T-Cell Activation Signaling

Natural TCR-IS delivers full activation signaling, while bsAb-T cells undergo insufficient stimulation in the absence of costimulatory signaling. Several studies have demonstrated that adding Signal 2 to bsAb-mediated T cell activation can augment T-cell antitumor efficacy. One recent study suggested a TSAxCD28 bispecific antibody could enhance the artificial IS and significantly improve T-cell antitumor activity when combined with TSAxCD3 bispecific antibodies (67). Apart from tumor antigens (TAs), immune checkpoint is another feasible target for Signal 2 stimulating bsAbs. For example, a PD-L1/CD28 BiTE can co-activate T cells with a TA/CD3 BiTE and convert an immunosuppressive signal into a costimulatory one (68). Besides CD28, 4-1BB has also been utilized to provide Signal 2 to bsAb-T cells. It has been reported that the TA-4-1BB ligand fusion protein (TA-4-1BBL) effectively activated T cells and eradicated the tumor in mouse models under the combination with CD3-directed bsAbs (69). A second strategy to complement bsAb-T cells Signal 2 is to lead them to self-express costimulatory ligands on the T-cell surface. CD19 engager (ENG)-T cells expressing CD80 and 4-1BBL have been proven superior antitumor activity against leukemia compared with unmodified CD19 ENG-T cells (70). Lastly, Signal 2 supplied by CAR molecules is also a potential strategy. Both BiTEs and CARs were introduced into T cells to generate BiTE-CAR-T cells, which could express CARs and also secrete BiTEs. BiTE-CAR-T cells received Signal 2 from CAR and probably had a more TCR-mediated like IS as BiTEs participation, which simultaneously enhanced BiTE-CAR-T cell function and effectively eliminated tumors without systemic toxicity (71).

Besides TCR and costimulatory signaling, cytokine signaling is thought to supply Signal 3 for the full activation of T cells, which has been proved in clinical trials (72, 73). Hence, the introduction of Signal 3 into CAR design might contribute to optimal CAR-T cell function. For example, CAR-T cells expressing interleukin-7 and CCL19, which are essential for maintaining T-cell zones in lymphoid organs, displayed superior antitumor activity and prolonged survival in mouse model (74). The addition of Janus kinase/signal transducer and activator of tran-ions (JAK-STAT) signaling domain into CAR constructs has also demonstrated superior CAR-T cell *in vivo* persistence and antitumor effect in both solid and non-solid models (75). Therefore, complement of T cell activation signaling should be a feasible strategy to stimulate optimal T cell activation and enhance redirected T cell therapeutic efficacy.

#### Adjustment of T Cell Signaling Strength

Signaling cascades caused by T-cell activation signals significantly influence T-cell immune response by mobilizing various downstream molecules and second messengers. These molecules further drive T-cell differentiation into distinct phenotypes and regulate T-cell metabolism. Investigations have generally reported naïve CD4^+^ T-cell differentiation is determined by the strength of TCR signaling (76). Strong TCR signaling favors the generation of Th1 over Th2 cells while weak TCR signaling promotes Th2 cell differentiation (77–79). Single CD28 stimulation has been found to promote stable and polyclonal expansion of Treg cells when TCR signaling is absent (80). Additionally, the strength of TCR signaling and costimulatory signaling is essential to T-cell activation. Over strong TCR signaling is inclined to induce T cell anergy or apoptosis, while weak but continuous TCR signaling plus strong co-stimulation can support a sustained T cell activation (8). Therefore, appropriate intensity of T-cell activation signaling is crucial.

Exhaustion is one of the main obstacles for *in vivo* CAR-T cell persistence. Hyperactivation is a potential explanation for CAR-T cell exhaustion. To find optimal signal strength, the intracellular signal domain of CAR has been widely modified. A CAR design incorporating CD3ϵ cytoplasmic domain, which recruits the inhibitory Csk kinase to attenuate TCR signaling, showed prolonged persistence and enhanced antitumor activity (81). In costimulatory domain, a study prolonged CAR-T cell persistence *via* mutating a Grb2-interacting residue in CD28 intracellular domain (82). The transcriptional profile of CD28-mutated CAR-T cells resemble ICOS signaling, exhibiting reduced T-cell exhaustion (82). CAR’s affinity is also essential to signal strength. CAT CAR-T cells with a lower affinity than FMC63 CAR-T cells showed enhanced *in vivo* antitumor activity and prolonged persistence in clinical trials (83). Lowering the binding affinity of CD123 CAR-T cells also exhibited higher specificity in treatment of acute myeloid leukemia, suggesting further application in avoiding potential on-target, off-tumor effect (84). Therefore, an optimized strength of TCR signaling and costimulatory signaling could be an alternative strategy for enhanced efficacy and prolonged persistence of redirected T cell therapies.

#### Reprogramming of T-Cell Metabolism

After activation signaling delivery from IS, T cells rapidly shift their metabolism to adapt to the massive energy demands for proliferation and immune functionality (85, 86). Each T cell subset has its unique metabolic characteristics (86, 87). For example, the metabolism of activated effector T cells is similar to cancer cells, which mainly depends on aerobic glycolysis to fuel their vigorous bioenergetic needs (11). However, long-lived memory cells have a relatively low metabolic need and generate ATP mainly through oxidative phosphorylation (OXPHOS) and fatty acid oxidation (88, 89). In addition, memory T cells have more mitochondrial mass and mitochondrial spare respiratory capacity, which allows them to swiftly switch to a high aerobic glycolysis mode after encountering target antigens (90, 91). By analyzing complete responding patients with chronic lymphocytic leukemia after CAR-T cell therapy, it revealed that CAR-T cells from these patients possessed memory-like characteristics and were enriched in memory-related genes (92). The transcriptomic profiling of anti-CD19 CAR T cells from patients with large B cell lymphomas revealed that patients who achieved complete response had three-fold higher frequencies of memory signatures expressing CD8^+^ T cells than patients with partial response or progressive disease (93). Meanwhile, memory T cells yielded optimal anti-tumor effects after transferring to a heightened metabolism mode *in vivo* (94). Therefore, CAR-T cell products with higher proportions of central memory T (Tcm) cells should display longer clinical efficacy and persistence than other T cell subsets *in vivo*. By this point, inducing T cell differentiation toward memory subsets by taking advantage of their metabolic characteristics should be another potential strategy to enhance the efficacy and prolong the persistence of redirected T cells.

Reprogramming T cell metabolism to modify T cell differentiation can be achieved by the regulation of activation signaling in the IS. Each costimulatory signaling has its own metabolic favor to T cells. For instance, CD28 co-stimulation is required for T cells to increase their glycolytic rate in response to activation, inducing naïve T cell differentiation toward effector T cells (95). CAR-T cells containing 4-1BB signaling domain showed memory phenotype metabolism tendency with increased mitochondrial biogenesis and central memory subsets, and had enhanced *in vitro* persistence (96). Similarly, supply of 4-1BB signaling in BiTE-T cells should be also a promising strategy for reprogramming BiTE-T cell metabolism.

Alternatively, the other candidates for reprogramming T cell metabolism are metabolic immune checkpoints (87). The glutaminolysis pathway plays a key role in immune metabolism reprogramming (97). It has been proven that blockade of both T cell and cancer cell glutamine metabolism by glutamine antagonism can suppress cancer cell survival but markedly upregulate T cell oxidative metabolism to a long-lived, highly-activated phenotype (98). Transient inhibition of Glutaminase may enhance CAR-T cell function and long-lasting cell survival *in vivo* (99). Therefore, reprogramming of T cell metabolism by directing T cell fate to memory subsets may be a potent strategy to yield enhanced efficacy and prolonged persistence for redirected T cell therapies.

## Conclusion

**Figure 4:**
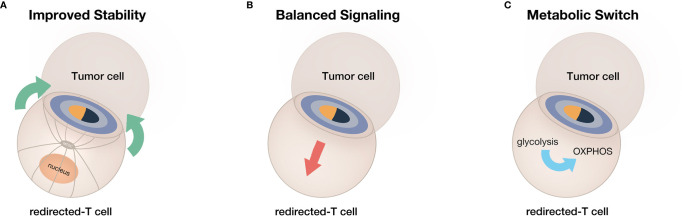
A summary of current strategies for obtaining enhanced efficacy and prolonged persistence from redirected T cells. **(A)** The improvement of IS stability. **(B)** The adjustment of IS-mediated signaling. **(C)** The switch of T-cell metabolism toward memory phenotype.

CAR or bsAb-redirected T-cell therapy has been regarded as the most promising cancer immunotherapy. However, further improving the clinical outcomes of redirected T cell therapy is currently an urgent demand due to its limited efficacy and poor persistence. Therefore, based on inspiration from IS generated by natural TCR, we discussed optimized strategies to boost the efficacy and improve the persistence of redirected T cells, including improved stability, balanced signaling, and reprogramming metabolism (**Figure 4**). By emulating the elaborate and sophisticated process of natural T cell activation, the therapeutic potential of redirected T cells can be fully explored. In conclusion, in-depth understanding of natural T-cell immune recognition, activation, and function is profound for optimized artificial receptor design and improved redirected T cell functionality.

## Author Contributions

YG, FL, and YC wrote, critically reviewed, and edited the manuscript. All authors contributed to the article and approved the submitted version.

## Funding

This work was supported by the National Natural Science Foundation of China (81730045, 81870375), Shanghai Rising-Star Program (18QA1401000), and Open Research Fund of State Key Laboratory of Genetic Engineering, Fudan University (SKLGE1911).

## Conflict of Interest

The authors declare that the research was conducted in the absence of any commercial or financial relationships that could be construed as a potential conflict of interest.
